# Polymodal Transient Receptor Potential Vanilloid (TRPV) Ion Channels in Chondrogenic Cells

**DOI:** 10.3390/ijms160818412

**Published:** 2015-08-07

**Authors:** Csilla Szűcs Somogyi, Csaba Matta, Zsofia Foldvari, Tamás Juhász, Éva Katona, Ádám Roland Takács, Tibor Hajdú, Nóra Dobrosi, Pál Gergely, Róza Zákány

**Affiliations:** 1 Department of Anatomy, Histology and Embryology, Faculty of Medicine, University of Debrecen, Debrecen H-4032, Hungary; E-Mails: matta.csaba@med.unideb.hu (C.M.); foldvarizsofia@gmail.com (Z.F.); juhaszt@anat.med.unideb.hu (T.J.); katona.eva@anat.med.unideb.hu (E.K.); takacs.roland@med.unideb.hu (A.R.T.); hajdu.tibor@anat.med.unideb.hu (T.H.); dobrosi.nora@med.unideb.hu (N.D.); 2 Department of Veterinary Preclinical Sciences, School of Veterinary Medicine, Faculty of Health and Medical Sciences, University of Surrey, Guildford, Surrey GU2 7XH, UK; 3 Cell Biology and Signalling Research Group of the Hungarian Academy of Sciences, Department of Medical Chemistry, Research Centre for Molecular Medicine, Faculty of Medicine, University of Debrecen, Debrecen H-4032, Hungary; E-Mail: gpal@med.unideb.hu

**Keywords:** mechanical loading, heat stimulation, cartilage formation, chondrocyte, high density culture, micromass, TRPV

## Abstract

Mature and developing chondrocytes exist in a microenvironment where mechanical load, changes of temperature, osmolarity and acidic pH may influence cellular metabolism. Polymodal Transient Receptor Potential Vanilloid (TRPV) receptors are environmental sensors mediating responses through activation of linked intracellular signalling pathways. In chondrogenic high density cultures established from limb buds of chicken and mouse embryos, we identified TRPV1, TRPV2, TRPV3, TRPV4 and TRPV6 mRNA expression with RT-PCR. In both cultures, a switch in the expression pattern of TRPVs was observed during cartilage formation. The inhibition of TRPVs with the non-selective calcium channel blocker ruthenium red diminished chondrogenesis and caused significant inhibition of proliferation. Incubating cell cultures at 41 °C elevated the expression of TRPV1, and increased cartilage matrix production. When chondrogenic cells were exposed to mechanical load at the time of their differentiation into matrix producing chondrocytes, we detected increased mRNA levels of TRPV3. Our results demonstrate that developing chondrocytes express a full palette of TRPV channels and the switch in the expression pattern suggests differentiation stage-dependent roles of TRPVs during cartilage formation. As TRPV1 and TRPV3 expression was altered by thermal and mechanical stimuli, respectively, these are candidate channels that contribute to the transduction of environmental stimuli in chondrogenic cells.

## 1. Introduction

Articular cartilage is a unique tissue with a low density of chondrocytes, where this sole cell type is capable of secreting and maintaining the abundant cartilaginous extracellular matrix (ECM). Since one of the key roles of articular cartilage is to distribute mechanical load and absorb shock generated during physical activity between opposing bones, chondrocytes are inherently adapted to the demands imposed by mechanical stimuli. Therefore, despite their low metabolic activity, chondrocytes communicate extensively with their environment partly through the dynamically changing ECM and respond to a range of mechanical and biochemical stimuli. Articular cartilage is a bradytroph tissue that does not contain blood vessels and is supplied with nutrients partially from the synovial fluid lubricating its surface [1]. In fact, the normal internal milieu of articular cartilage might be regarded as non-physiological compared to other tissues [2]. Towards the subchondral bone, oxygen tension is gradually decreasing to as low as 1%, and lactic acid, the end product of the anaerobic metabolism of chondrocytes, accumulates, resulting in a particularly acidic environment with pH around 6.5 [3]. Mature chondrocytes are well adapted to these conditions but rarely proliferate once they became fully mature. This unfavourable property of its resident cells manifests in a very limited capacity for spontaneous regeneration of articular cartilage in case of joint injury or degeneration. Despite the partial functional restitution (*i.e.*, autologous chondrocyte implantation, autologous mesenchymal stem cell transplantation or osteochondral autografts), none of the currently available options for the repair of damaged cartilage have proved to be satisfactory so far in articular cartilage tissue engineering [4]. Clearly, there is a strong need to obtain more information about the differentiation process of hyaline cartilage to improve our knowledge for cell-based therapies in the field of cartilage regeneration.

During joint movements, articular tissues are exposed to shear and compressive stress; these mechanical stimuli are important not only in the mature tissue, but they are also indispensable to the development of both the articular surface and the underlying zones. For instance, lack of mechanical stimulation during *in vitro* chondrogenesis of mesenchymal stem cells commonly leads to terminal hypertrophy of chondrocytes [5]. Appropriate frequency and strength of the mechanical load are also essential for mature chondrocytes to maintain proper lubrication, nourishment and removal of metabolic waste products via the synovial fluid [1,2,6].

Intensive physical activities may cause local elevation of temperature in articular tissues; however, little is known about the impact of temperature change on cartilage. Pritchett described that in a normal hip joint the temperature of synovial fluid generally increases 1 °C after 20 min and 2 °C after 60 min of walking, although other factors, such as body mass, age, exercise type and intensity have not been taken into consideration [7,8,9]. Although this is a relatively understudied area and available data are limited, we can assume that heat may alter the metabolic activity of chondrocytes together with the mechanical properties of the ECM [10,11,12].

Various plasma membrane receptors and ion channels are implicated to be responsible for mediating environmental stimuli in articular chondrocytes [13,14,15]. Polymodal Transient Receptor Potential Vanilloid (TRPV) ion channels are promising candidates to transduce diverse stimuli (thermal, mechanical stress, acidity and aniso-osmolarity) for chondrocytes. These channels are characterised by six putative transmembrane spans (TM) and a cation-permeable pore region between TM5 and TM6. The NH_2_ and COOH termini are located intracellularly, vary in length, and contain different numbers of functional domains and motifs. These ion channels, assembled as homo- or heterotetramers, are sensitive to a remarkable range of stimuli [16,17].

Several studies reported the presence of certain TRPVs in synovial joints. According to Szabo and his colleagues, TRPV1 has a role in the development of chronic arthritis [18]. Eight channels of the TRP superfamily, including TRPV1, have been identified in osteoarthritic cartilage tissue samples [19]. Expression of other vanilloid receptors, such as TRPV4, TRPV5 and TRPV6, has also been reported in articular chondrocytes [20]. The role of TRPV4 in cartilage is of particular interest, since this channel seems to be a positive regulator of Sox9, a master gene of chondrogenic differentiation [21]; gain-of-function mutations of this ion channel can cause severe musculoskeletal diseases [22,23] and it seems to be involved in mediating the metabolic activities of mature cartilage [24].

This study describes the presence and possible functions of TRPV receptors during *in vitro* chondrogenesis. We applied avian and murine high density cultures, wherein spontaneous cartilage differentiation occurs. These models display the physiological course of chondrogenesis, during which limb bud-derived chondroprogenitor mesenchymal cells undergo condensation and nodule formation and differentiate into chondroblasts and chondrocytes, producing and secreting cartilage-specific ECM components including collagen type II and aggrecan. We identified several vanilloid receptors at mRNA level and analysed their expression pattern after thermal and mechanical stimulation. Based on our results, we propose that the presence and precise regulation of their expression pattern may play a role during cartilage formation.

## 2. Results

### 2.1. mRNA Expression Profiling of TRPV Ion Channels in Chicken and Mouse Tissue Samples

**Figure 1:**
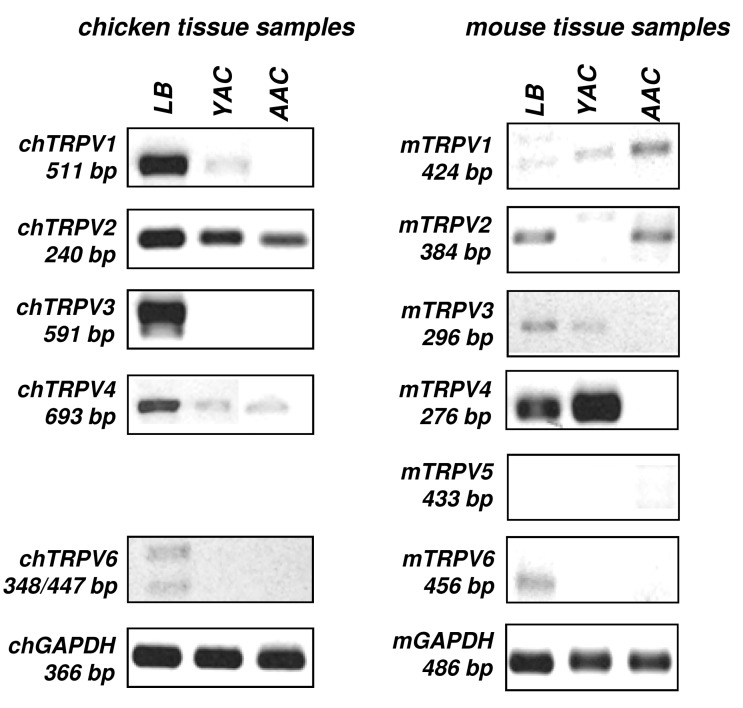
Conventional PCR analysis of TRPV expression in chicken (ch) and mouse (m) tissue samples. TRPV mRNA expression was monitored in chicken and mouse limb buds (LB), articular cartilage derived from young (YAC), and adult animals (AAC). Glyceraldehyde 3-phosphate dehydrogenase (GAPDH) was used as an internal control. Chicken TRPV5 expression was not analysed due to unknown sequence data. Representative data of three independent experiments.

First, we screened the mRNA expression of all types of vanilloid receptors in chicken and mouse cartilage samples taken from chicken embryonic limb buds (LB), one-day-old, young chicken articular cartilage (YAC), adult articular cartilage (AAC), mouse embryonic limb buds (LB), two-day-old, young mouse articular cartilage (YAC), adult mouse articular cartilage (AAC). The designed primer pairs were tested in positive control tissues such as brain and kidney (Figure S1). As the chicken TRPV5 sequence was not available in GenBank, we were unable to design specific primer pairs (Table 1). As seen in Figure 1, mRNAs of almost all TRPVs were found to be expressed in both chicken and mouse distal limb buds (LB), where the majority of the cells are chondroprogenitor cells. The age of the analysed limb bud tissues was identical to those from which micromass cell cultures are established. In the articular cartilage of young animals (one or two-day-old animals; YAC; Figure 1), where the dominant cell types are young and still proliferating chondrocytes, the expression spectrum of TRPVs seemed to decrease. An even narrower subset of TRPV mRNAs was detectable in articular cartilage samples of adult animals (AAC; Figure 1) in which a well-developed zonal architecture and mature, non-dividing chondrocytes are present [25].

**Table 1:** Nucleotide sequences, amplification sites, GenBank accession numbers, amplimer sizes and PCR reaction conditions for each chicken primer pair are shown. * annealing temperature (°C).

Chicken Gene	Primer	Primer Sequences (5′→3′)	GenBank Accession No.	*	Amplimer Size (bp)
Transient Receptor Potential Vanilloid type 1 (TRPV1_chick)	forward	TTCGTTCACTCTTTGCTCCTC (1633–1653)	**NM_204572.1**	58	511
	reverse	TGCTCACAGTTTCTCCCATCA (2143–2123)			
Transient Receptor Potential Vanilloid type 2 (TRPV2_chick)	forward	CCCTTGGAGTCACCTTACC (547–565)	**XM_004946687.1**	54	240
	reverse	CTTCCCAGTCTTTGCATCT (786–768)			
Transient Receptor Potential Vanilloid type 3 (TRPV3_chick)	forward	CCCCTCAATTCACTCCTGC (2794–2812)	**XM_004946676.1**	60	591
	reverse	GGAAAGGCATTCACCACCA (3384–3366)			
Transient Receptor Potential Vanilloid type 4 (TRPV4_chick)	forward	TCGCCGAGAAGACGGGAAAC (733–752)	**NM_204692.1**	60	693
	reverse	GGCGGTTCTCAATCTTGCTGTT (1425–1404)			
Transient Receptor Potential Vanilloid type 6 (TRPV6_chick)	forward	CATGTAGCTGCCTTGTATGA (806–825) or (429–448)	**XM_004938143.1**	52	348
	reverse	TGATCTTGGTCCCTCTTTG (1153–1135) or (875–857)	**XM_004938142.1**		447
Aggrecan core protein (ACAN_chick)	forward	CAATGCAGAGTACAGAGA (435–452)	**NM_204955.2**	54	429
	reverse	TCTGTCTCACGGACACCG (863–846)			
Collagen II (COL2A1_chick)	forward	GGACCCAAAGGACAGACGG (1191–1209)	**NM_204426.1**	59	401
	reverse	TCGCCAGGAGCACCAGTT (1591–1574)			
SRY (sex determining region Y)-box 9 (SOX9_chick)	forward	CCCCAACGCCATCTTCAA (713–730)	**NM_204281.1**	54	381
	reverse	CTGCTGATGCCGTAGGTA (1093–1076)			
Glyceraldehyde 3-phosphate dehydrogenase (GAPDH_chick)	forward	CTGCCCAGAACATCATCCCA (656–675)	**NM_204305.1**	58	366
	reverse	CACGGTTGCTGTATCCAAACTCAT (1021–998)			

### 2.2. mRNA Expression Profiling of TRPV in Embryonic Limb Bud-Derived Micromass Cultures

**Figure 2:**
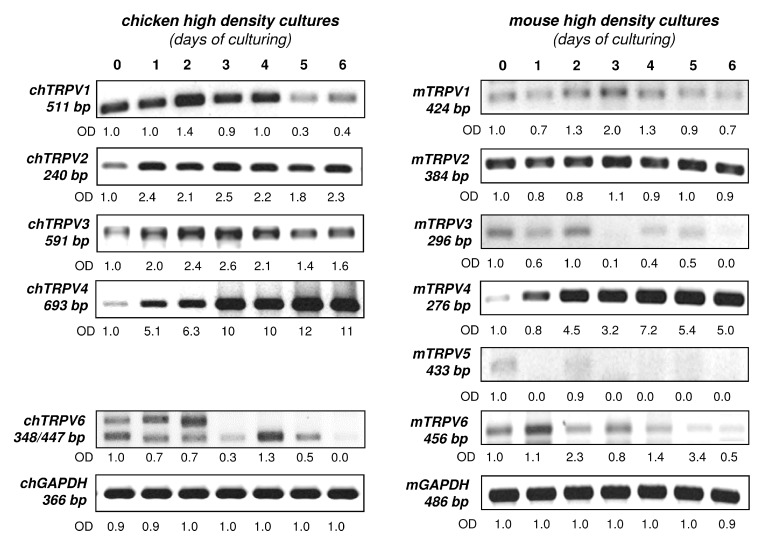
mRNA expression of TRPVs in chicken (ch) and mouse (m) HD cultures. GAPDH was applied as an internal control. Numbers below gel images represent integrated densities of signals determined using ImageJ 1.46; data were normalised to the value detectable on the earliest day of culturing, *i.e.*, day 0 (1.0) where applicable. Chicken TRPV5 expression was not analysed due to unpublished sequence data. GAPDH was used as an internal control. Representative data of three independent experiments.

In the following set of experiments, we screened the mRNA expression of various TRPVs in high density micromass cell cultures (HD). The advantage of the avian model over the murine model is that it is cost effective and yields higher initial cell number for the experiments. On the other hand, the amino acid sequences of TRPV proteins are different from the mammalian TRPV orthologues resulting in different responses to pharmacological modulations. Therefore, we decided to compare the mRNA expression of chicken and mouse HD cultures during the seven-day-culturing (Figure 2). The mRNA expression pattern of TRPV4 was the most distinct as it exhibited a steady elevation in both models. TRPV2 showed a relatively constant expression in both chicken and mouse HD cultures. In both experimental models TRPV1 and TRPV3 displayed a weaker signal towards the end of the culturing period. This was consistent in three independent chicken and mouse culture series and also confirmed by other TRPV1 and TRPV3 primers that were designed to amplify different sequences. Chicken TRPV6 primer pairs recognised two transcript variants with two different product lengths (348 bp for XM_004938143.1 and 447 bp for XM_004938142.1). Murine HD cultures displayed both TRPV5 and TRPV6 mRNAs whose expression pattern gradually decreased by the end of the culturing period. (The chicken and mouse TRPV GenBank IDs and the sequences of designed primers are listed in Table 1 and Table 2.)

**Table 2:** Nucleotide sequences, amplification sites, GenBank accession numbers, amplimer sizes and PCR reaction conditions for each mouse primer pair are shown. * annealing temperature (°C).

Mouse Gene	Primer	Primer Sequences (5′→3′)	GenBank Accession No.	*	Amplimer Size (bp)
Transient Receptor Potential Vanilloid type 1 (TRPV1_mouse)	forward	CTCTTACAACAGCCTGTATTCC (2130–2151)	NM_001001445.2	59	424
	reverse	ACAGTTGCCTGGGTCCTC (2553–2536)			
Transient Receptor Potential Vanilloid type 2 (TRPV2_mouse)	forward	CTTTGCTGTAGCCCTAGTAAGC (2007–2028)	NM_011706.2	59	384
	reverse	CACCACCAGTAACCATTCTCC (2390–2370)			
Transient Receptor Potential Vanilloid type 3 (TRPV3_mouse)	forward	AGCAGAACTCCACCTACCC (1998–2016)	NM_145099.2	58	296
	reverse	TTTCCATTCCGTCCACTT (2293–2276)			
Transient Receptor Potential Vanilloid type 4 (TRPV4_mouse)	forward	TCTGTCTCGCAAGTTCAAGG (1314–1333)	NM_022017.3	59	276
	reverse	GGCTGATAGTAGGCGGTGA (1589–1571)			
Transient Receptor Potential Vanilloid type 5 (TRPV5_mouse)	forward	TCCGAGATGCCAACCGTAC (1108–1126)	NM_001007572.2	59	433
	reverse	GCCATTAGCCAGCAGAAGC (1540–1522)			
Transient Receptor Potential Vanilloid type 6 (TRPV6_mouse)	forward	GCTGGCTGATGGCTGTGGT (1773–1791)	NM_022413.4	63	456
	reverse	GGCGGATGCGTTGTCTGTT (2228–2210)			
Glyceraldehyde 3-phosphate dehydrogenase (GAPDH_mouse)	forward	TGGCAAAGTGGAGATTGTTG (161–180)	NM_001289726.1	58	486
	reverse	GTCTTCTGGGTGGCAGTGAT (646–627)			

### 2.3. Effect of Ruthenium Red, a General TRPV Inhibitor, on Chondrogenesis

**Figure 3:**
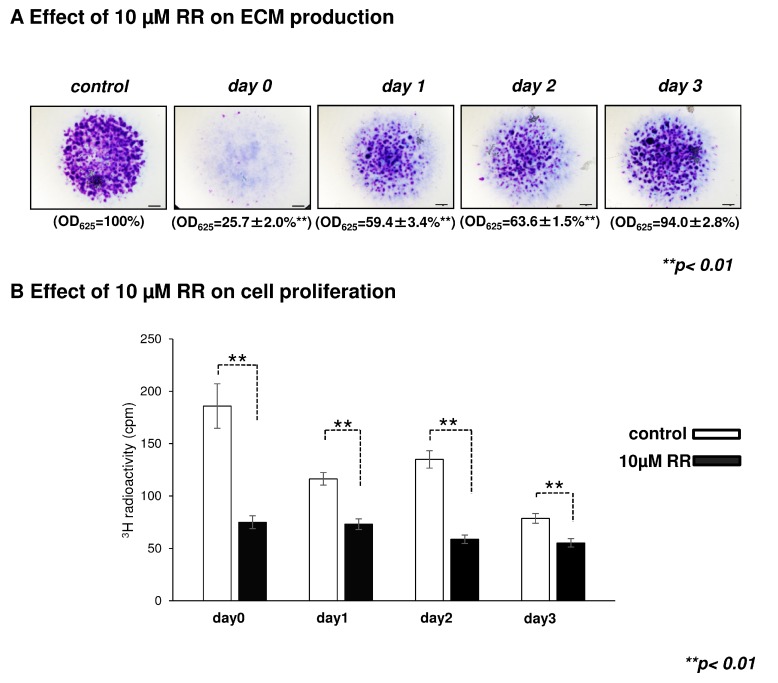
The effect of ruthenium red (RR) treatment on cartilage matrix production and cellular proliferation. (**A**) The cartilage matrix of six-day-old micromass cultures were stained with acidic dimethyl-methylene blue (DMMB) after treating the colonies with 10 µM RR for 24 h on days 0, 1, 2 or 3 of culturing. Representative photomicrographs out of four independent experiments are shown. Original magnification was 2×. Scale bar, 1 mm. Optical density (OD_625_) was determined in samples containing toluidine blue (TB) extracted with 8% HCl dissolved in absolute ethanol. Data are expressed as mean ± SEM. Asterisks (**) represent significant difference compared to control cultures (** *p* < 0.01). Displayed values are from one representative assay out of four independent experiments; (**B**) RR significantly decreased cellular proliferation rate (^3^H-T) in chicken micromass cultures. Statistically significant differences are marked by asterisks (** *p* < 0.01). Data shown are from one representative experiment out of four.

Ruthenium red (RR), a broad-spectrum calcium channel inhibitor, was administered for 24 h on different days of culturing at a concentration of 10 µM. We measured its effect on matrix production and proliferation. Application of RR on the first three days of culturing significantly reduced the amount of metachromatically stained proteoglycan-rich ECM (Figure 3A) and also caused a significant decline in the number of proliferating chondrogenic cells (Figure 3B).

### 2.4. The Effect of Heat Treatment on Chondrogenesis

**Figure 4:**
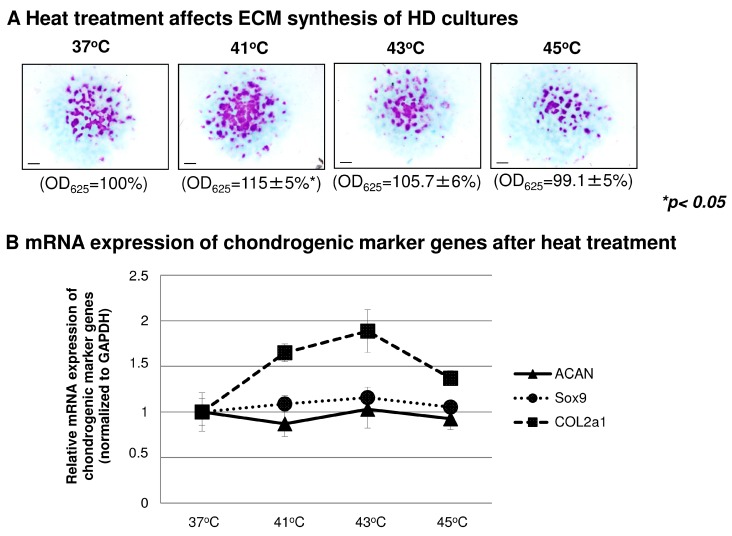

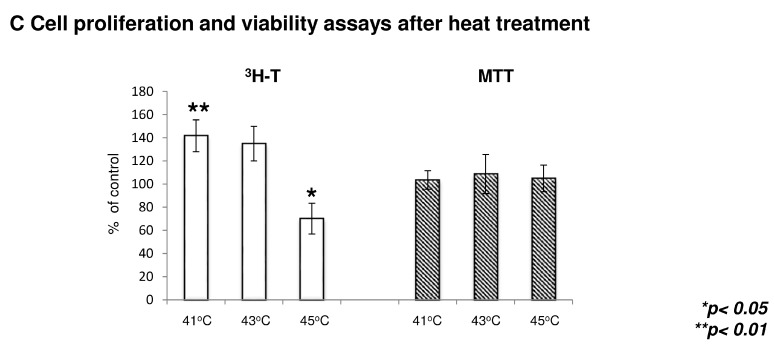
The effect of heat treatment on cartilage matrix production, chondrogenic marker gene expression and cellular proliferation. (**A**) Metachromatic cartilage matrix in micromass cultures after applying different heat stimuli (41, 43 and 45 °C for 30 min). Metachromatic cartilage areas in six-day-old cultures were visualised with DMMB dissolved in 3% acetic acid. Representative photomicrographs of 13 independent experiments are shown. Original magnification was 2×. Scale bar, 1 mm. Optical density (OD_625_) was determined in samples containing TB extracted with 8% HCl dissolved in absolute ethanol. Data are expressed as mean ± SEM. Asterisks represent significant difference compared to control cultures (* *p* < 0.05). Data shown are the average of 13 independent experiments; (**B**) Conventional RT-PCR analysis of chondrogenic markers genes (ACAN; aggrecan core protein, Sox9, and COL2a1; collagen type II). Chicken micromass cultures were exposed to 41, 43 or 45 °C for 30 min, and the mRNA expression pattern was monitored 90 min after the heat treatment. The expression of marker genes were normalised to GAPDH. The graph displays the average expression levels detected in 3 independent experiments; (**C**) Effect of heat treatment on cellular proliferation rate (^3^H-T) and mitochondrial activity (MTT). Statistically significant proliferation rate of cells in cultures that were stimulated *vs.* control cultures are marked by asterisks (* *p* < 0.05 and ** *p* < 0.01). Data shown are the average of seven independent experiments.

Joint movements and inflammation can raise the local temperature in the joints, which may affect cartilage matrix production, cell proliferation and cell metabolism. We aimed to check whether heat treatment may influence cartilage matrix production in HD cultures. Matrix production was approximated with metachromatic staining. As seen in Figure 4A, heating to 41 °C significantly enhanced the amount of sulphated glycosaminoglycans (GAGs) (to 115% ± 5%). Interestingly, however, higher temperatures did not seem to affect cartilage ECM production. A slight decrease was observed in the case of 45 °C treated groups, but the mean of 13 independent semi-quantitative TB staining results indicated non-significant change. Next, we aimed to explore the expression of Sox9, the master gene of chondrogenesis, and the genes encoding cartilage ECM components (*i.e.*, collagen type II and aggrecan core protein). Their transcript levels were monitored with RT-PCR analysis 90 min after the heat treatment. We failed to detect any prominent changes in the mRNA expressions of these markers, and only moderate alterations were observed as seen in Figure 4B.

The increased amount of proteoglycan-rich ECM might be the result of either enhanced proliferation or metabolic activity. While heat treatment did not alter the metabolic activity of chondrogenic cells, the proliferation rate of cultures treated with 41 °C for 30 min significantly increased compared to untreated control cultures. On the other hand, the 45 °C treated group showed a significantly reduced proliferation rate (Figure 4C).

Heat stimulation can induce heat shock protein (HSP) expression [26,27], influencing various cellular functions, including proliferation. We analysed the expression levels of HSP47, HSP70, and HSP90 following heat stimuli, but none of the investigated HSPs showed any significant change in their mRNA expression pattern as determined by RT-PCR (Figure S2).

### 2.5. Heat Treatment and its Effect on High Density (HD) Cultures in the Context of TRPVs

It is well known that polymodal TRPV ion channels are thermosensitive [16]. If the channels are affected during the heat treatment, *i.e.*, exposure to temperatures that are relatively close to the channel activation threshold, the mRNA expression of these receptors could become altered. To test this hypothesis, we applied heat stimuli ranging from 37 to 45 °C (37, 41, 43 and 45 °C) on HD cultures and monitored changes in the mRNA expression of TRPVs over a 24 h period.

**Figure 5:**
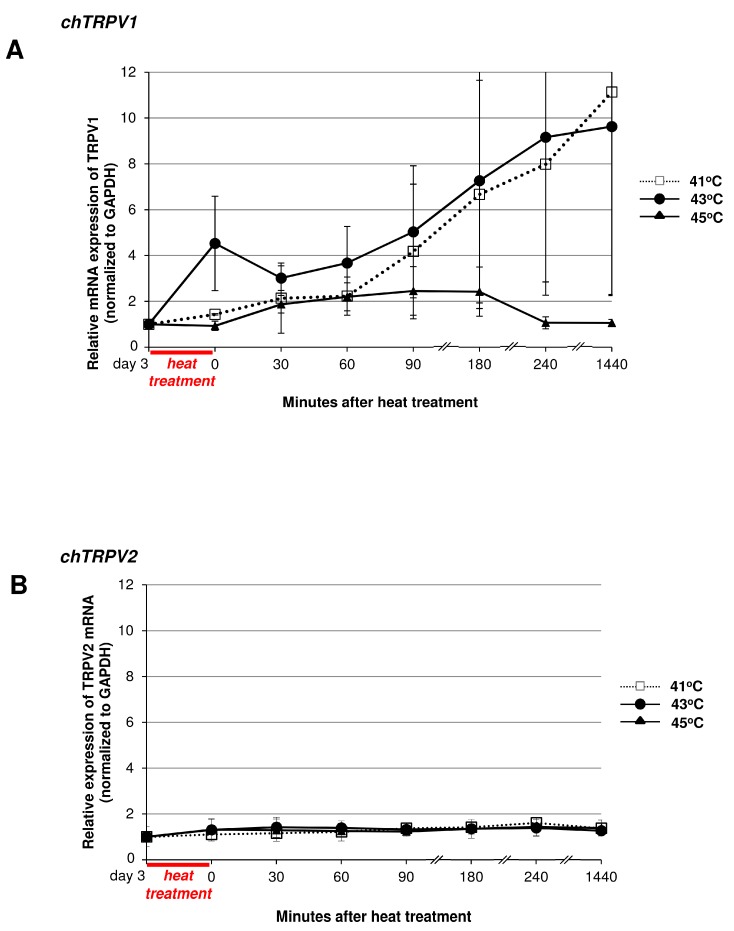

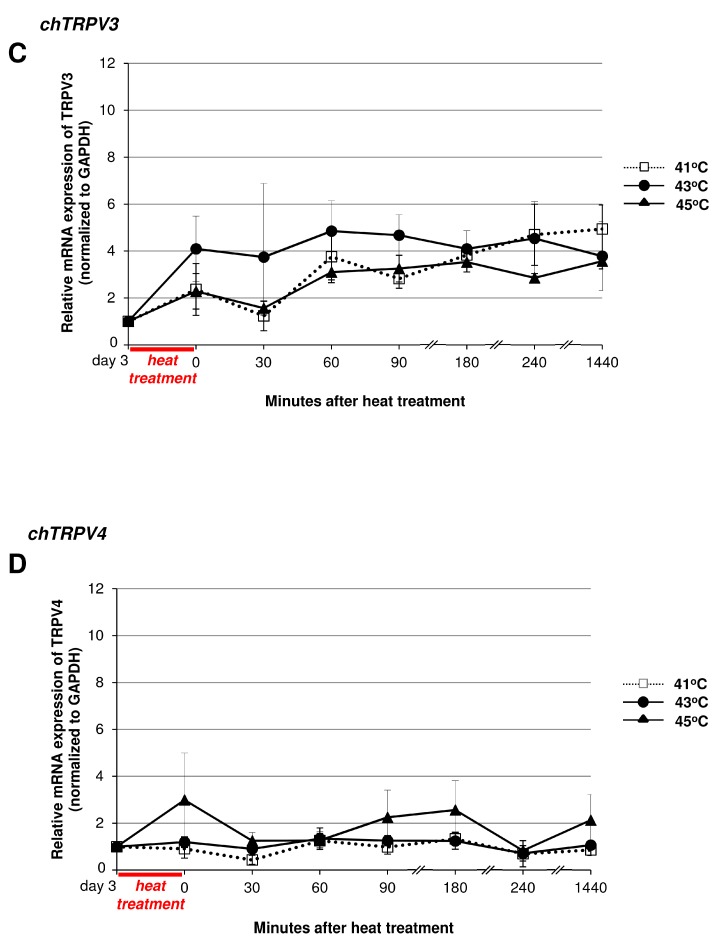
RT-PCR analysis of chicken TRPV1-4 (**A**–**D**) mRNA expression patterns following heat treatment. Chicken micromass cultures were kept at 41, 43 and 45 °C for 30 min and the mRNA expression pattern was monitored for 24 h. TRPV mRNA expression was normalised to GAPDH. The graph displays the average expression of three independent experiments (mean ± SEM).

TRPV1 is a non-selective cation channel with an activation threshold of around 41.5 °C [28,29], but this value may slightly vary among species and different cell types, and also depends on the pH of the environment [30]. We detected an almost ten-fold increase of mRNA expression in the 41 and 43 °C treated groups, while the 45 °C treated group showed only a moderate increase and, by the end of the 24 h period, the expression values returned to the control level (Figure 5A). TRPV2 is characterised by a far higher thermal activation threshold of around 52 °C [16,31] and accordingly, the applied temperature values did not affect the mRNA expression pattern of TRPV2 during the 24-h monitoring period (Figure 5B). In this way, the unchanged expression pattern of TRPV2 might serve as an internal control since those channels that are not activated by the applied temperature ranges seem to be unaffected at mRNA level. TRPV3 and TRPV4 mRNA expression levels showed slight (4-fold and 2-fold, respectively) elevation in response to heat stimuli (Figure 5C,D). The activation threshold of these ion channels is close to the applied heat stimuli (TRPV3 is around 33 °C and TRPV4 is approx. 27 °C [16]), and it seems to affect the mRNA expression pattern of these membrane receptors (Figure 5C,D).

### 2.6. Mechanical Load in the Context of TRPVs

**Figure 6:**
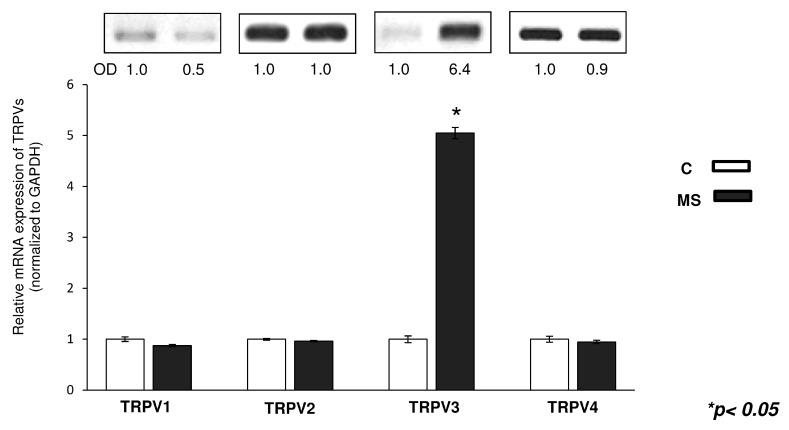
mRNA expression of TRPVs in control (C) and mechanically stimulated (MS) chicken HD cultures on the third day of culturing. Numbers below gel images represent integrated densities (OD) of signals determined with ImageJ 1.46. The average values of integrated band densities of 4 independent experiments are visualised by the graph. The expression levels of TRPV transcripts were normalised to GAPDH.

During joint movement, mechanical load is the most physiological stimulus affecting cartilage *in vivo*, and we have already described that mechanical stimulation enhances chondrogenesis via the PKA/CREB-Sox9 and PP2A pathways in chicken micromass cultures [15]. Since certain TRPVs are suggested contributing to mechanosensation in various cell types [32], we aimed to find out whether cyclic uniaxial mechanical load influenced the mRNA expression level of TRPV receptors. As seen in Figure 6, the mRNA expression of TRPV3 exhibited significant increase following mechanical load as revealed by semi-quantitative RT-PCR analyses. The mRNA expression of other TRPVs did not show any significant alterations upon mechanical load (Figure 6).

## 3. Discussion

### 3.1. TRPV Ion Channels Are Expressed during in Vitro Chondrogenesis

In this study, we aimed to investigate the presence and putative role of various polymodal TRPV ion channels in chicken and mouse chondrogenic cells and articular cartilage. Most of the TRPVs were identified at mRNA levels in chondrogenic micromass cultures as well as articular cartilage samples. While TRPV1, 2, 3, 4 and 6 were present at the mRNA level in chicken HD cultures, mouse HD cultures exhibited all TRPVs. Both experimental models displayed similar mRNA expression patterns in the case of TRPV1, 3 and 4 during the investigated period. TRPV1 and 3 mRNAs showed a peak around culturing days two and three in both species. Of note, the final commitment of chondrogenic cells takes place specifically on these culturing days in HD cultures [33,34]; therefore, we assume that these ion channels might influence the process of chondrogenesis. To support this hypothesis, we treated the HD cultures with RR for a 24 h, starting from different culturing days (day 0, 1, 2, 3). Application of RR during the first days of culturing significantly decreased the amount of metachromatic ECM (Figure 3A), hampering chondrogenic differentiation. As we observed the strongest inhibition when RR was applied on day 1 of culturing, we suppose that TRPVs may play a role in the early events of cartilage formation, such as nodule formation and expansion of cell number. Indeed, a remarkable suppression of proliferation was observed in RR treated cultures (Figure 3B).

To the best of our knowledge, there are no specific pharmacons that were proved to be reliable in modulating the activity of avian TRPV orthologues. Specific agonists (e.g., capsaicin in case of TRPV1, camphor in case of TRPV3) or antagonists are not applicable in case of chicken TRPV ion channels, since these agents affect mammalian orthologues only as a result of strong evolutionary pressure [35,36,37,38]. First, we decided to check whether capsaicin (cap), resiniferatoxin (RTX) or capsazepine exert any effect on the chondrogenesis in high density cultures. We applied the semi-quantitative toluidine blue dye extraction assay to evaluate the amount of metachromatically stained cartilage matrix. However, as seen in Supplementary Figure S5, there were no significant changes after the treatments and the results were inconsistent (Figure S5). The only TRPV channel blocker that we could apply was the aspecific but widely accepted ruthenium red [16].

Expression of multiple TRPVs can reflect on either a redundancy in their function or a combination of the various subunits in the tetrameric receptors. It has already been reported that vanilloid receptors might form heterotetramers, *i.e.*, TRPV1 with TRPV2, or TRPV5 with TRPV6, [39,40]; moreover, Yao and colleagues identified heteromeric TRP channels composed of subunits from 3 different TRP subfamilies [41]. The similar expression pattern of TRPV1 and TRPV3 raises the possibility that these vanilloid receptors assemble into heterotetramers in chondroprogenitor cells. A similar receptor composition was demonstrated by Smith and colleagues describing heterotetrameric TRPV1–TRPV3 complexes in human dorsal root ganglia [42].

In both chicken and mouse model systems TRPV4 displayed a gradually increasing mRNA expression in HD cultures as cartilage differentiation was in progress, implying that this channel is necessary for certain functions of more mature chondrocytes. Indeed, it has already been reported that TRPV4 had an expression pattern similar to chondrogenic marker genes in ATDC5 and C3H10T1/2 chondrogenic cell lines during *in vitro* chondrogenesis [21]. Recently, Bateman and his lab provided evidence of TRPV4 expression during *in vivo* chondrogenesis [43]. Several articles reported that point mutations of this ion channel can be directly linked to skeletal malformations of varying severity [22,44,45]. Besides its presence in chondrogenesis, TRPV4 has also been suggested as a mechano- and osmosensor in adult articular cartilage [14,46]. The expression pattern of TRPV4 has been screened by some laboratories in relation to the course of chondrogenesis, but none of them investigated it in HD cultures along with other TRPV receptors.

We observed a switch in the expression pattern of TRPVs during chondrogenesis. The precise role of TRP channels during development has not been determined, but it is well documented that some ion channels have distinct but vital roles in embryonic and adult stages of life [47]. For instance, NMDA (*N*-methyl-d-aspartate) receptors regulate neuronal cell migration during development, while they act as key regulators for neuronal excitation in adults [47]. Preclinical studies have identified that TRPs are involved in hereditary neuropathies together with neuronal disorders [48]. Since these polymodal channels are expressed in primary afferent nociceptors and pain sensing neurons, they also contribute in the perception of various physical and chemical stimuli [49,50], hence they are in the focus of pain research. On the other hand, there is growing evidence that TRP channels have other novel functions beside nociception, for instance their expression is altered in cancer cells compared to normal ones [51]. These proteins are also involved in cell proliferation, apoptosis, migration [52,53] and invasion [51]. There are also studies describing their function in neural progenitor differentiation [54,55]. Therefore, it is possible that these TRP channels have distinct roles in mesenchymal cells and in differentiated chondrocytes. Our data indicate that chondrogenic mesenchymal cells express TRPV1 and 3 at the beginning of culturing when migrating mesenchymal cells form cell aggregates, proliferate and establish cartilage nodules. This might imply that TRPV1 and 3 are more important in mesenchymal cell migration and proliferation during chondrogenesis and indeed, there are several articles describing vanilloid ion channels as key figures of cell migration [52,53]. By the time of chondrogenic differentiation chondroblasts downregulate these TRPV channels and at the same time enhance TRPV4 expression. A possible explanation to support this theory is that once mature chondrocytes occupy their lacunae and acquire a sedentary lifestyle, the presence and a possible function of TRPV4 in mechano- and osmotic signal transduction may become vital.

We investigated the expression pattern of TRPV1, 3 and 4 proteins in chicken chondrogenic cells; however, we had to abandon analysing protein expression in these cultures. As the amino acid sequence of the avian TRPV1 orthologue displays approx. 68% identity with the murine TRPV1 ion channel [35], finding a chicken-specific TRPV1 antibody was a challenge. The majority of the commercially available antibodies are raised against those parts of the mammalian TRPV1 sequence that hardly overlap with the avian orthologue. We applied two polyclonal antibodies: ab72431 (Abcam Ltd., Cambridge, UK), specific for the chicken N-terminal; and ab74813 (Abcam), recognising the third cytoplasmic loop of avian and mammalian TRPV1 (Figure S3A). As it is demonstrated in Supplementary Figure 3B, several bands appeared on Western blots for both antibodies besides the predicted size (96 kDa). After many technical modifications that involved experimenting with longer blocking periods, we were able to gain clearer signals, but some prominent bands around e.g., 130, 85 and 70 kDa still remained visible for both antibodies.

However, at least some of these bands may still correspond to TRPV1 proteins; according to the literature several TRPV1 splice variants exist [56,57,58,59,60], and there are multiple sites for post-translational modifications, which cause alterations in the molecular weight [60,61,62,63]. We also tested other antibodies against TRPV3 and TRPV4 in both cases, but showed very similar results, *i.e.*, multiple bands (Figure S4). Nonetheless, the appearance of multiple signals does not necessarily mean that these bands would represent non-specific signals only, given the fact that there are for example posttranslational modifications and splicing variants of TRPVs. The bands which appear at higher molecular weights than the predicted might be present as the result of glycosylation [45], while the bands that are visible at lower molecular weights than predicted (e.g., around 75 kDa in case of TPRV4) can represent splice variants [64]. Overall, the above mentioned problems—appearance of several bands, resulting either from aspecific binding, post-translational modifications, or species differences—seem to be general in case of all TRPV western blots applied in chicken HD cultures. Therefore, using Western blotting as the main approach to confirm the presence of TRPV proteins in chicken chondrogenic cells is challenging.

### 3.2. Thermal and Mechanical Stimuli Alter Polymodal TRPV Ion Channel Expression in Micromass Cultures

As we failed to unequivocally detect the presence of TRPV1, 3 and 4 proteins, we decided to find indirect evidence for the presence of these channels. To this end, we aimed to reveal possible connections between the alterations of the microenvironment surrounding differentiating chondrocytes and TRPV receptors. We found that 30 min 41 °C heat stimuli on culturing day three increased cell proliferation and enhanced metachromatic matrix deposition. There is limited data about the positive effect of heat stimulation on skeletal tissues, for instance, *in ovo* temperature manipulation influences embryonic motility and growth of limb tissues in the chick [65]. Lovejoy and his group observed that temperature regulates limb length in homeotherms by directly modulating cartilage growth [66]. They reported that chondrocyte proliferation and extracellular matrix volume strongly correlate with tissue temperature in metatarsals cultured without vasculature *in vitro* [66]. Furthermore, Chen and colleagues demonstrated that periodic heat shock (41 °C for 1 h) accelerated chondrogenic differentiation of human mesenchymal stem cells in pellet culture [67]. Together with our results, these data clearly demonstrate that moderate temperature stimulations positively influence cartilage development and suggest the role for thermosensitive TRP channels as regulators of chondrogenesis. While the positive effect of heat stimulation in normal or osteoarthritic adult articular cartilage homeostasis has also been suggested by some laboratories [68,69], other authors argue that heat treatment alone does not increase proteoglycan (PG) synthesis; however, in those cases, the experimental conditions (stimulation length, culture system or temperature values) were different from our study [12,70]. Nevertheless, temperature is a fundamental environmental variable during joint movement and, despite the fact that it seems to influence chondrogenesis and cartilage metabolism, it is still a relatively understudied field affecting cartilage behaviour [11].

Since TRPVs are polymodal ion channels activated by several environmental stimuli, we decided to screen their mRNA expression pattern after heat stimulation. Alterations in the mRNA expressions of certain TRPV channels seem to be related to the activation range of the given ion channel. For instance, TRPV1 can be characterised by a temperature threshold around 41.5 °C [28,29]. After the 30 min heat treatment, TRPV1 expression seems to be strongly modulated by temperatures 41 and 43 °C, temperature ranges close or equivalent to the activation threshold of this channel. TRPV3 with an activation range of around 33 °C is the next vanilloid receptor displaying moderate mRNA expression changes, whereas TRPV4, whose activation is close to room temperature, seems to be less affected by the heat stimulus. TRPV2 (≥52 °C), whose activation range is far from the applied stimulus, or heat shock proteins display no changes in their mRNA expression patterns. It might be the case that during the heat stimulus certain enhancers influence the expression of heat sensitive TRPVs [71]. It is worth mentioning that the activation threshold of TRPV1 can change under certain ambient conditions, such as low pH of the microenvironment. During inflammation or in the deeper zones of articular cartilage, the acidic microenvironment might lead to the activation and sensitisation of TRPV1 channel at lower temperatures [59]. In fact, the heteromeric assembly and the high redundancy between TRPVs might explain why the genetic deletion of certain TRPVs does not result in significant deficit in the animals [72].

There is accumulating data about TRPVs [73,74], and especially about TRPV1, suggesting their role in cell proliferation. In certain cases TRPV1 stimulation triggers proliferation [75,76], but overactivation of TRPV1 ion channels and the resulting increased calcium influx can be toxic to the cells [77]. Nevertheless, the applied heat stimuli did not exert any toxic effect in our experiments. As the TRPV1 mRNA expression decreased in case of the 45 °C groups compared to the other cultures, the altered presence or function of TRPV1 could account for the decreases in proliferation and metachromatic staining.

Proper mechanical load is essential for the healthy structure of articular cartilage in foetal and mature synovial joints [14,15,78,79]. As a result of joint load there are fluctuations in the osmotic environment of chondrocytes eliciting calcium influx through mechanosensitive ion channels [80]. TRPV4 can act as a mechano- and osmosensor in articular chondrocytes, and it is also regarded as a regulator of chondrogenic differentiation [81,82]. Accordingly, we investigated the mRNA expression pattern of TRPV ion channels in mechanically loaded HD cultures. Surprisingly, only the mRNA expression of TRPV3 rose significantly after mechanical load. There are no articles describing if TRPV3 is linked to mechanotransduction of cells, however, we find it interesting that the mRNA of TRPV3 increased significantly, compared to the rest of the ion channels. Some articles mention that the mechanical stretch inhibits adipogenesis and stimulates osteogenesis of adipose stem cells [83,84], another study describes that TRPV3 channel activation suppresses adipogenesis [85]. Since the mesenchymal cells in the micromass cultures have the potential to differentiate towards the osteogenic, chondrogenic and adipogenic lineages [86], it might be the case that there is a connection between mechanical stimulation and adipogenesis through TRPV3 mRNA upregulation.

Our results reflect on the diversity of the expressed TRPV channels in developing chondrocytes and provide evidence of their functionality during *in vitro* chondrogenesis. Clearly, further investigations are required to prove their role during *in vivo* cartilage formation and maintenance. Nonetheless, the polymodal nature of their activation and the possibility of heterotetramer formation increase the difficulties and the complexity of their investigation.

## 4. Experimental Section

### 4.1. Tissue Sample Collection

Limb buds were collected from four-day-old chicken embryos after Ross hybrid chicken eggs were incubated in a commercial hatcher at 39 °C, under 85% humidity in our laboratory. Tissue samples from freshly hatched chicks and from chickens of 12 weeks of age were kindly provided by the laboratory of János Oláh (Centre for Agricultural Sciences, University of Debrecen, Debrecen, Hungary). Animals were treated according to the regulations defined in the licence obtained from the University of Debrecen, Committee of Animal Research (XXVI-KÁT/2013) and were sacrificed by cervical dislocation. For mouse limb bud-derived HD cultures NMRI (Naval Medical Research Institute) laboratory strain mice were mated overnight and mating was confirmed by the presence of a vaginal plug (considered as day 0 of gestation). On day 11.5 of gestation, mouse embryos were removed from the uterine horns, washed in sterile calcium and magnesium-free phosphate buffered saline (CMF-PBS) several times, and then the distal limb buds were harvested. Tissue samples from two-day-old and 12-week-old mice were also collected. As the tissues/organs (limb buds, articular cartilage from knee joints) were removed they were immediately snap frozen in liquid nitrogen and stored at −80 °C until use. Animals were treated according to the regulations defined in the licence obtained from the University of Debrecen, Committee of Animal Research (11/2010/DE MÁB) and were sacrificed by cervical dislocation.

### 4.2. Chicken and Mouse HD Primer Cell Cultures

Chicken and mouse HD cultures were prepared as described previously [13,87]. Briefly, distal parts of four-day-old Ross hybrid chicken embryo limb buds (Hamburger-Hamilton stages 22–24) were isolated and digested with 0.25% trypsin-EDTA (Sigma-Aldrich, St. Louis, MO, USA; pH 7.4) solution at 37 °C for one hour. After terminating digestion with an equal amount of foetal bovine serum (FBS; Gibco, Gaithersburg, MD, USA), cells were filtered through a 20-μm plastic filter unit (Millipore, Billerica, MA, USA) and seeded onto the surface of cell culture dishes at a concentration of 1.5 × 10^7^ cells/mL. These chondrifying mesenchymal cells were allowed to attach to the surface for two hours, and then were grown in Ham’s F12 medium (Sigma-Aldrich) supplemented with 10% FBS and kept at 37 °C in a CO_2_ incubator (5% CO_2_ and 80% humidity). The medium was changed on every second day or after treatments. The day of seeding was considered as day 0. For some colonies, ruthenium red (RR) (Sigma-Aldrich) as a general TRPV inhibitor was added to the medium on certain culturing days for a single 24 h application at a final concentration of 10 μM (stock: 10 mM dissolved in water).

Mouse HD cultures were established from cells obtained from the distal limb buds of 11.5-day-old NMRI laboratory mouse embryos. The isolation and culturing of these HD cultures were similar to chicken HD cultures with minor modifications [86,88,89].

### 4.3. mRNA Expression Analysis Followed by Reverse Transcription Polymerase Chain Reaction

Tissue samples, HD colonies from each day of culturing (from day 0 to day 6), as well as control and heat stressed cultures on the 3rd day of culturing were collected, frozen in liquid nitrogen, and stored at −80 °C until use. Then samples were dissolved in TRIzol (Applied Biosystems, Foster City, CA, USA), and following addition of 20% RNase-free chloroform (Sigma-Aldrich) samples were centrifuged at 10,000× *g* for 15 min at 4 °C. Total RNA-containing samples were incubated in 500 μL RNase-free isopropanol at −20 °C for 1 h, total RNA was dissolved in nuclease-free water (Promega, Madison, WI, USA) and stored at −80 °C [86,87].

The assay mixture for reverse transcriptase reactions contained 1000 ng RNA, 1 μL RNase inhibitor 0.8 µL 25× dNTP Mix (100 mM), 2 μL RT random primers, 1 μL MultiScribe Reverse Transcriptase, 2 μL 10× RT Buffer filled up to 20 μL with Nuclease Free Water (Promega) (High Capacity RT kit; Applied Biosystems). cDNA was transcribed at 37 °C for 2 h.

Amplification of specific cDNA sequences was carried out using specific primer pairs designed by Primer Premier 5.0 software (Premier Biosoft, Palo Alto, CA, USA) based on chicken and mouse nucleotide sequences published in GenBank. Primers were ordered from Integrated DNA Technologies, Inc. (IDT; Coralville, IA, USA). The specificity of custom-designed primer pairs was confirmed *in silico* by using the Primer-BLAST service of NCBI. Nucleotide sequences of forward and reverse primers and reaction conditions are shown in Table 1 and Table 2. Amplifications were performed by GoTaq DNA Polymerase (Promega) according to the manufacturer’s protocol in a Labnet MultiGene™ 96-well Gradient Thermal Cycler (Labnet International, Edison, NJ, USA), as follows: initial denaturation for 2 min at 95 °C, followed by 35 cycles (denaturation for 30 s at 95 °C, primer specific annealing temperature for 30 s, extension for 30 s at 72 °C) and then 5 min for final extension at 72 °C. PCR products were analysed by electrophoresis in 1.2% agarose gel containing ethidium bromide. Finally, optical density of PCR product signals was determined by using ImageJ freeware (Image Processing and Analysis in Java, NIH, Bethesda, MD, USA) version1.46.

### 4.4. Heat Treatment of Chicken HD Cultures

Chicken HD cultures were incubated at 41, 43 and 45 °C for 30 min on the 3rd day of culturing. Samples were taken for RT-PCR at different time periods: 0, 30, 60, 90, 180 and 240 min and one day (24 h) after the heat shock. On the 6th day of culturing, these colonies were stained with DMMB and TB.

### 4.5. Assessment of Chondrogenic Differentiation by Metachromatic Staining

For the qualitative and semi-quantitative evaluation of cartilage matrix production, six-day-old cell cultures from different experimental groups (heat treated and mechanically loaded groups) were stained with DMMB (pH 1.8; Sigma-Aldrich) and with TB (pH 2; Reanal, Budapest, Hungary) metachromatic dyes as previously described [13,90]. Microphotographs of metachromatic cartilaginous nodules were taken with an Olympus DP72 camera on a Nikon Eclipse E800 microscope; image acquisition was performed by Spot Advanced software (version 4.6; Diagnostic Instruments, Inc., Burroughs, Sterling Heights, MI, USA). Optical density of extracted TB of different experimental groups was determined in three cultures of each experimental group in 13 independent experiments.

### 4.6. Measuring Cell Proliferation with ^3^H-Thymidine Labelling and Mitochondrial Activity with MTT Assay

^3^H-thymidine incorporation serves as a method for determining the rate of cell proliferation. Immediately after heat stress on the 3rd day of culturing medium containing 1 mCi/mL ^3^H-thymidine (diluted from methyl-^3^H-thymidine; 185 GBq/mmol; American Radiolabeled Chemicals, Inc., St. Louis, MO, USA) was added to the cell cultures for a 16-hour-long period. In the case of 24-hour-long ruthenium red treatment ^3^H-thymidine was kept on the colonies for 24 h. After washing twice with phosphate buffered saline (PBS), proteins were precipitated with ice-cold 5% trichloroacetic acid, washed with PBS again and placed into special, opaque 96 well microtiter plates (Wallac, PerkinElmer Life and Analytical Sciences, Shelton, CT, USA). Then these plates were placed into an exsiccator containing phosphorous pentoxide in order to absorb moisture. Prior to measurements, 50 µL scintillation solution (Maxilight; Hidex, Turku, Finland) was added to each well and radioactivity was measured by a liquid scintillation counter (Chameleon; Hidex, Turku, Finland). Data shown for heat treatment are the average of seven independent experiments; for RR treatment, data of one representative experiment is shown out of four.

For the investigation of cellular viability mitochondrial activity was detected with MTT-assays performed immediately after heat shock on day 3, as it was described previously [90]. Three-day-old HD cultures that had not received heat stress were used as controls. Measurements were carried out in four samples of each experimental group in seven independent experiments. The results were statistically analysed with Student’s *t*-test.

### 4.7. Mechanical Loading of HD Cultures

Chicken HD cultures grown in six-well plates were subjected to uniaxial cyclic compressive force (0.05 Hz, 600 Pa) on culturing days two and three for 30 min using a custom-made mechanical stimulator unit. For a detailed description of the stimulator unit please see Juhasz *et al.* [15]. Mechanical stimulation was carried out on both culturing days two and three for 30 min in a CO_2_ incubator (37 °C, 5% CO_2_, 80% humidity). Control cultures were grown under identical culture conditions without mechanical stimulation. Mechanically stimulated samples were collected for PCR analysis.

### 4.8. Statistical Analysis

Statistical significance of differences was evaluated using Student’s *t-*test. Differences were considered significant when *p* was less than 0.05 or 0.01 and marked by asterisks * (if *p* < 0.05) or ** (if *p* < 0.01) on the graphs. Results are expressed as mean ± SEM values.

## 5. Conclusions

In summary, we proved that both chicken and mouse primary chondrogenic cells express nearly the full palette of TRPV ion channels. A switch in the mRNA expression of TRPV1 and TRPV4 during the course of chondrogenesis suggests a distinct role of these channels in young and mature chondrocytes. Non-selective inhibition of TRPV receptors with RR resulted in attenuated cartilage formation and cell proliferation. The thermo- and mechanosensitive ion channels TRPV1 and TRPV3 responded with an altered mRNA expression to heat and/or mechanical stimulation, respectively, reflecting on the functionality of the expressed receptors. Considering the high sequence and functional similarity, the possibility of heterotetramer formation, and that multiple TRPVs are expressed by both undifferentiated and mature chondrocytes, a simultaneous analysis of the TRPV family members in future studies of cartilage formation is proposed.

## Supplementary Materials

Supplementary materials can be found at http://www.mdpi.com/1422-0067/16/08/18412/s1.

## Abbreviations

AAC: adult articular cartilage
YAC: young articular cartilage
CREB: cAMP response element-binding protein
CMF-PBS: calcium and magnesium-free phosphate buffered saline
DMMB: dimethyl-methylene blue
ECM: extracellular matrix
FBS: foetal bovine serum
GAG: glycosaminoglycan
GAPDH: glyceraldehyde 3-phosphate dehydrogenase
HSP: heat shock protein
HD: high density
LB: limb bud
MTT: 3-(4,5-dimethylthiazol-2-yl)-2,5-diphenyltetrazolium bromide
NMRI: Naval Medical Research Institute
PBS: phosphate buffered saline
NMDA: *N*-methyl-d-aspartate
PG: proteoglycan
PKA: protein kinase A
RR: ruthenium red
TB: toluidine blue
TRPV: transient receptor potential receptor vanilloid
TM: transmembrane
