# Supplementary material for: Polymodal Transient Receptor Potential Vanilloid (TRPV) Ion Channels in Chondrogenic Cells
Source: Int J Mol Sci. 2015 Aug 7;16(8):18412–38. doi: 10.3390/ijms160818412 (PMC4581253; doi:10.3390/ijms160818412)
Supplement: Supplementary file 1 [file ijms-16-18412-s001.pdf]

Supplementary Information

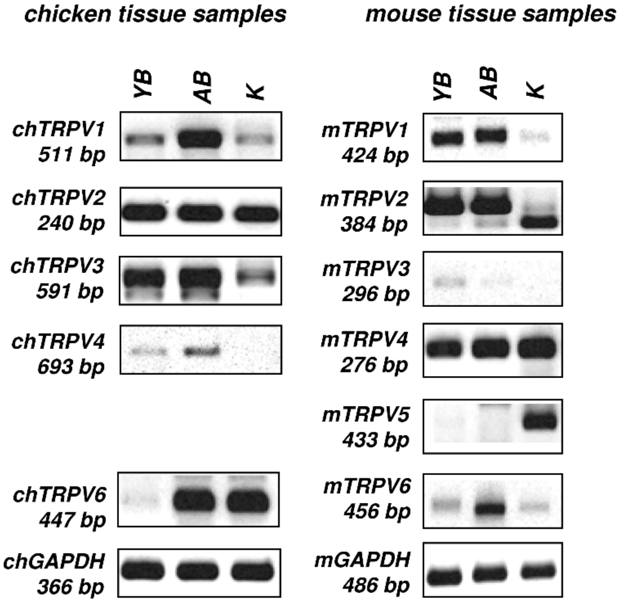

Figure S1. TRPV primers were tested in tissue samples proved to express the investigated TRPV channels, *i.e.*, one-day-old chicken brain (*YB*), adult chicken brain (*AB*), chicken kidney (*K*), two-day-old mouse brain (*YB*), adult mouse brain (*AB*), mouse kidney (*K*).

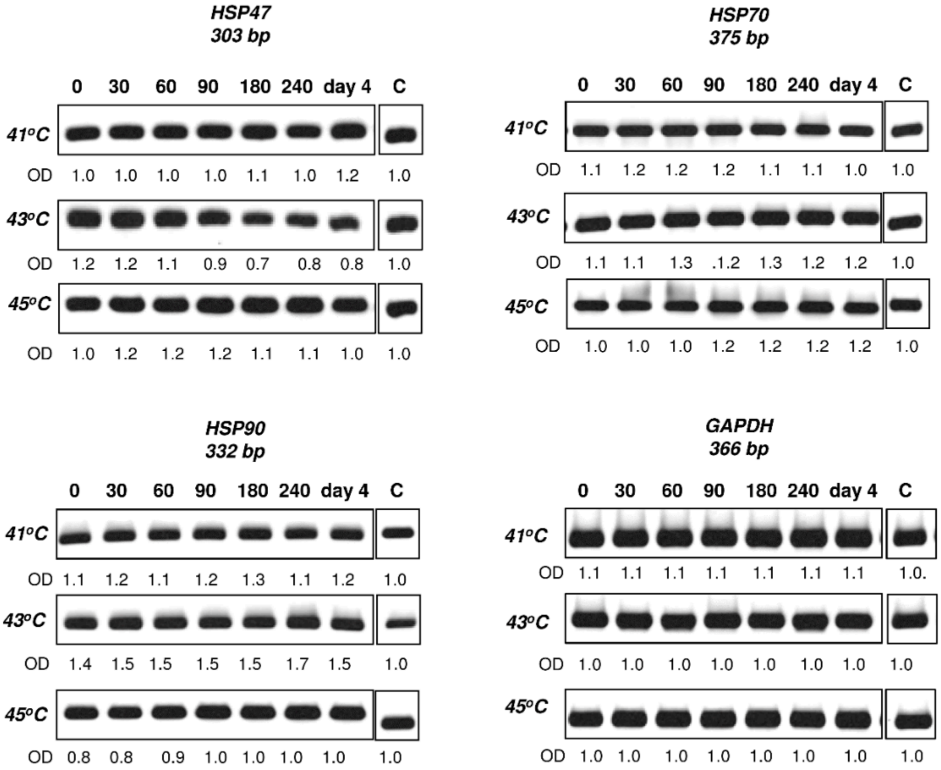

Figure S2. Chicken micromass cultures were exposed to 41, 43 or 45 °C for 30 min, and the mRNA expression pattern of heat shock proteins (HSP47, HSP70, HSP90) was monitored for a 24-hour-long period (0, 30, 60, 90, 180, 240 min and 24 h). The expression of marker genes were normalised to control cultures (C). GAPDH was used as an internal control. Representative data of 3 independent experiments.

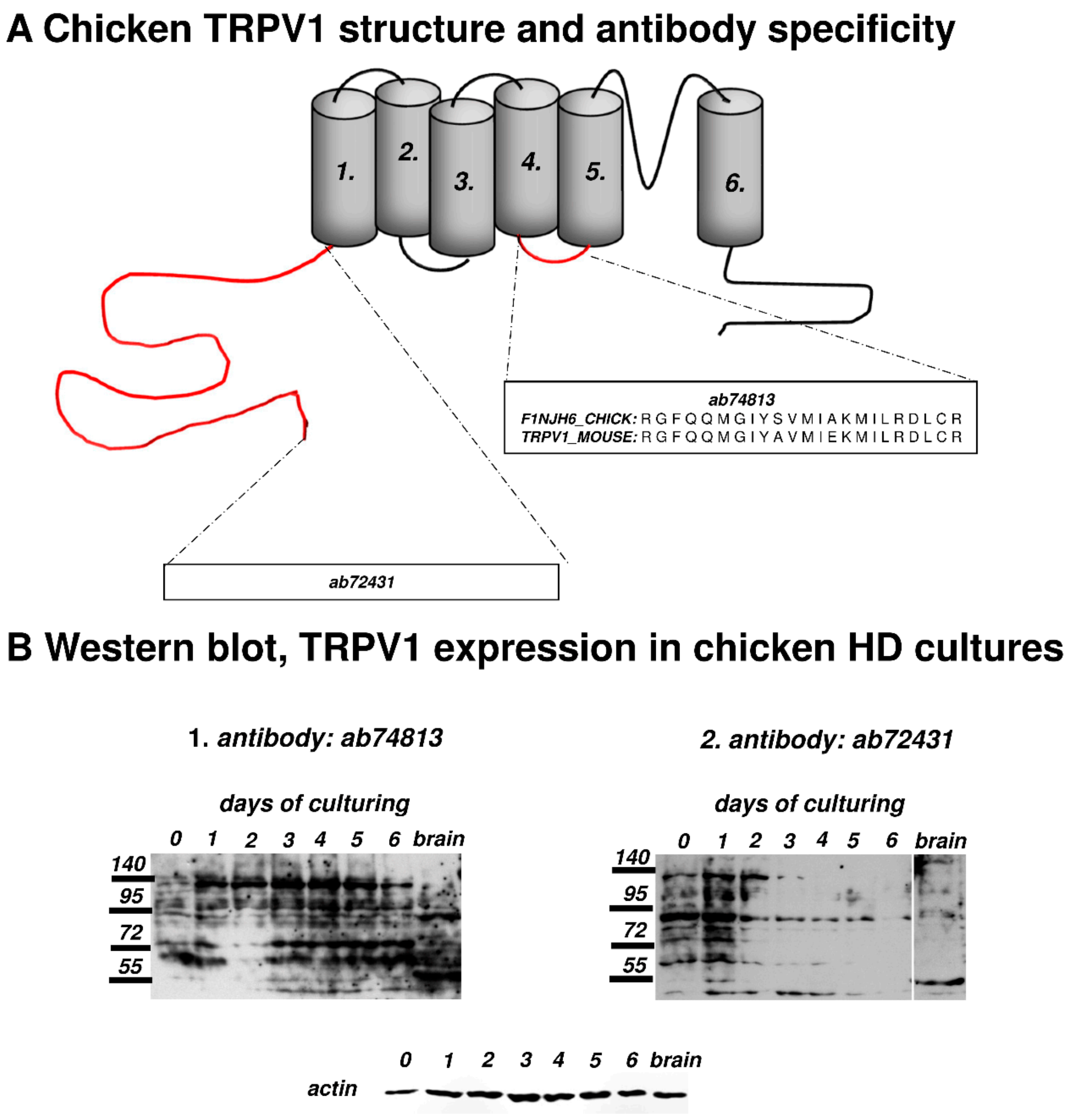

Figure S3. (**A**) Schematic representation of a TRPV1 receptor. The red segments display sequences against the applied antibodies (highlighted by the dotted lines) used in this study. ab72431 (Abcam) recognises the N-terminal sequence (red segment) of chicken TRPV1, while ab74813 (Abcam) recognises the third citoplasmic loop (red segments) of chicken and mammalian TRPV1 ion channels; (**B**) TRPV1 Western blot on chicken HD cultures (days 0–6) and chicken brain sample. Predicted molecular weight for chicken TRPV1 is 95–96 kDa. β-actin was used as an internal control.

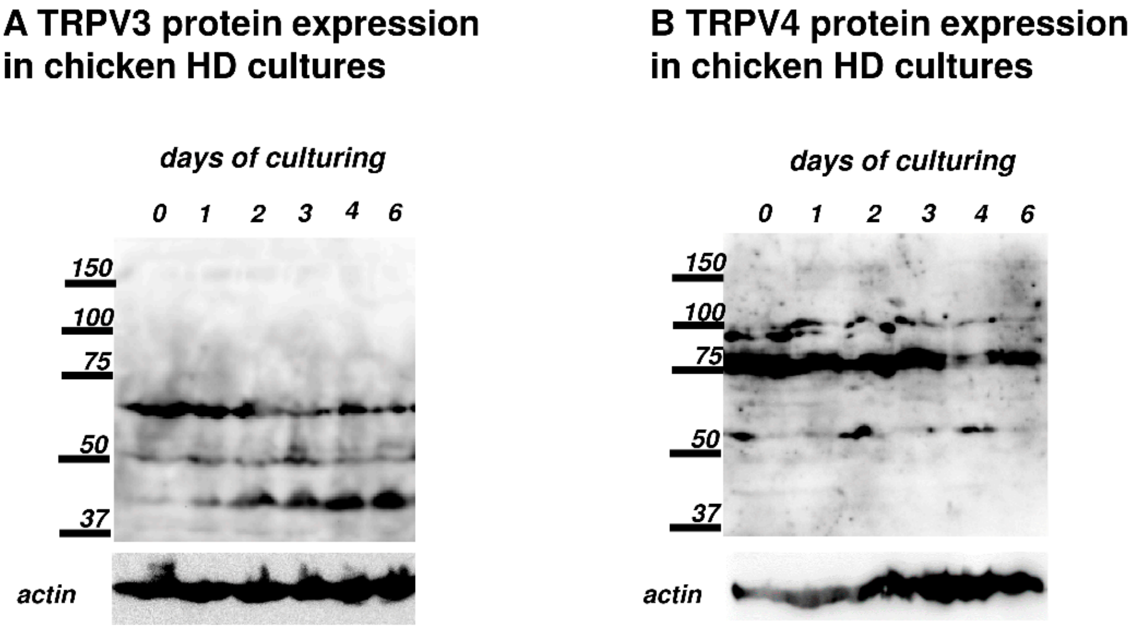

Figure S4. Protein expression of TRPV3 (**A**) and TRPV4 (**B**) in chicken HD cultures during the six-day-long culturing. For TRPV3, a polyclonal (NBP1-46342 Novus Biologicals, Cambridge, UK) and a monoclonal antibody (NBP2-12909 Novus Biologicals, Cambridge, UK) were tested. Whilst these antibodies recognise mammalian TRPV3, the extent to which they recognise the chicken sequence is unknown. For the polyclonal TRPV3 antibody, we did not observe any bands on the Western blots (data not shown). For the monoclonal antibody, whilst we did not detect bands at the predicted 90 kDa molecular weight specific to TRPV3, additional bands appeared below 75 kDa (A). For TRPV4 (predicted molecular weight is around 98 kDa), two antibodies were tested. Unfortunately, the one (ACC-134, Alomone, Israel) that should have recognised an extracellular loop of TRPV4 did not give any signals (data not shown). On the other hand, the antibody from Abcam (ab39260) showed three bands. These bands might be the result of alternative splicing (B). β-actin was used as an internal control.

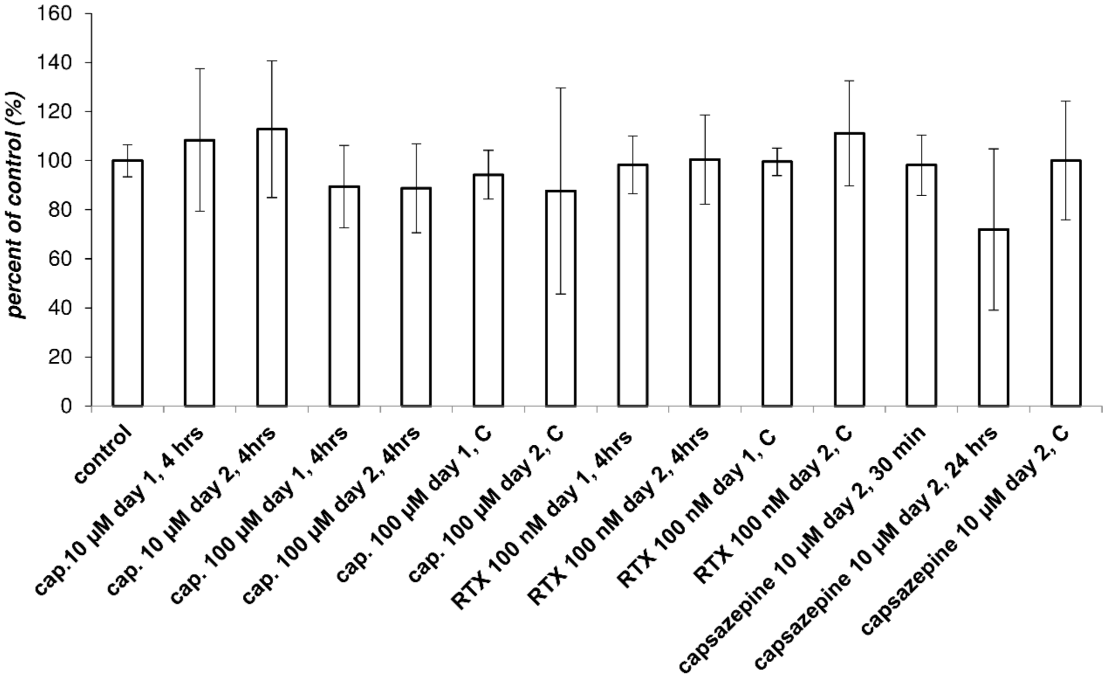

Figure S5. Chicken HD cultures were treated with TRPV1 agonists (capsaicin, cap; and resiniferatoxin, RTX) and a TRPV1 antagonist (capsazepine) at different time intervals and concentrations. On day 6 of culturing colonies were stained with toluidine blue, and then the dye was extracted with 8% HCl dissolved in ethanol. Measuring the absorbance and comparing it to the control provides a semi-quantitative method to evaluate the amount of metachromatically stained cartilage matrix. Optical density of extracted TB of different experimental groups was determined in 2 cultures of each experimental group in 6 independent experiments. Abbreviations: cap, capsaicin; RTX, resiniferatoxin; C, continuous treatment.
